# Supplementary material for: Causal Relationship between Aging and Anorexia Nervosa: A White-Matter-Microstructure-Mediated Mendelian Randomization Analysis
Source: Biomedicines. 2024 Aug 16;12(8):1874. doi: 10.3390/biomedicines12081874 (PMC11351342; doi:10.3390/biomedicines12081874)
Supplement: Supplementary file 1 [file biomedicines-12-01874-s001.zip › Supplementary Figure/Figure S8/Leaveoneout plot of MR analysis for TL to ubm-b-1504.pdf]

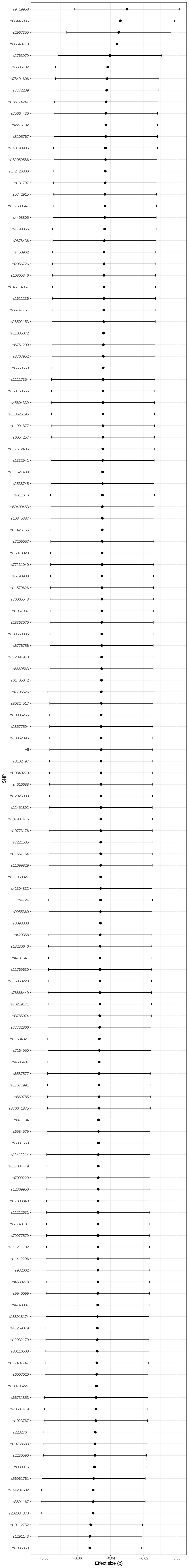
rs9419958
rs35446936
rs2967355
rs35640778
rs2763979
rs6536702
rs78491606
rs7772289
rs185174247
rs75664430
rs2276182
rs8105767
rs143190905
rs182059586
rs142426306
rs131797
rs5742915
rs117630647
rs4498805
rs7790856
rs9878436
rs450962
rs2056726
rs10805346
rs145114957
rs1611236
rs55747751
rs28502153
rs11085072
rs6751209
rs3767952
rs6659669
rs11117354
rs150150565
rs45604339
rs113525195
rs11991877
rs6054257
rs117512405
rs1332941
rs111527438
rs2538745
rs611646
rs59409453
rs10845387
rs11426156
rs7209057
rs16978028
rs77231040
rs6790988
rs11579626
rs76065543
rs1957937
rs28363070
rs139669835
rs6776756
rs112394943
rs6669563
rs61405042
rs7705526
rs80324517
rs10905255
rs28577594
rs13062095
All
rs8102497
rs10840270
rs4616688
rs12925933
rs12451892
rs137901416
rs10773176
rs7221585
rs11557154
rs11699829
rs111950327
rs41304832
rs4724
rs9955360
rs3093888
rs429358
rs13230646
rs4731541
rs11769630
rs116863223
rs76666449
rs76219171
rs3785074
rs77732866
rs11584821
rs7164950
rs4695407
rs6587577
rs17677991
rs869785
rs376641875
rs871134
rs6584579
rs6881568
rs12412214
rs117034449
rs7099229
rs12369950
rs17803849
rs11212631
rs61748181
rs79977579
rs141214782
rs11412296
rs932002
rs4530278
rs9940099
rs4743037
rs188918174
rs41269079
rs12932179
rs80116508
rs117407747
rs6007020
rs139795227
rs66731853
rs73581419
rs1023767
rs2282764
rs10768683
rs2230590
rs939916
rs56061761
rs144204502
rs3891167
rs202034370
rs10112752
rs1291143
rs1985369
SNP
−0.08
−0.06
−0.04
−0.02
0.00
Effect size (b)
